# Paeoniflorin attenuates sepsis-induced liver injury by reprogramming macrophage polarization via the TLR4/NF-κB pathway

**DOI:** 10.3389/fimmu.2025.1751550

**Published:** 2026-01-20

**Authors:** Zhiwei Rong, Baitian Li, Chunzheng Liu, Lijun Liao

**Affiliations:** 1 Postgraduate Training Base of Jinzhou Medical University, Shanghai East Hospital, Shanghai, China; 2 Department of Pain Management, Shanghai East Hospital, Tongji University School of Medicine, Shanghai, China; 3 Department of Emergency Medicine, The Second Affiliated Hospital of Anhui Medical University, Hefei, Anhui, China

**Keywords:** hepatoprotection, macrophage polarization, paeoniflorin, sepsis-associated liver injury, TLR4/NF-κB pathway

## Abstract

**Background:**

Sepsis-associated liver injury (SALI) increases mortality in critically ill patients but lacks targeted treatments. Although the natural compound Paeoniflorin shows anti-inflammatory and immunomodulatory potential, its specific function and mechanism in SALI remain unclear.

**Methods:**

A murine model of polymicrobial sepsis was established using cecal ligation and puncture (CLP). Male C57BL/6 mice were randomly allocated to Sham, CLP, CLP+Paeoniflorin (30, 60, 120 mg/kg), CLP+Paeoniflorin+TLR4 agonist (RS09 TFA), and Paeoniflorin-only control groups. Liver injury was assessed through serum ALT/AST measurements, histopathological evaluation, and TUNEL apoptosis assay. Hepatic inflammatory cytokine expression was quantified by qPCR. Macrophage polarization was analyzed via immunohistochemistry for F4/80, CD86 (M1), and CD206 (M2) markers. TLR4/NF-κB pathway activity was examined using Western blotting and immunohistochemistry. Transcriptomic profiling was performed through RNA sequencing and KEGG pathway analysis.

**Results:**

Paeoniflorin administration significantly attenuated CLP-induced elevations in serum ALT and AST levels in a dose-dependent manner, ameliorated histopathological liver damage, and reduced hepatocyte apoptosis. Treatment with Paeoniflorin substantially downregulated hepatic mRNA expression of pro-inflammatory cytokines (IL-6, TNF-α, IL-1β). Immunohistochemical analysis revealed that Paeoniflorin treatment was associated with a shift in macrophage marker expression, characterized by a reduction in cells co-staining for F4/80 and the classic M1 marker CD86, and an increase in cells co-staining for F4/80 and the classic M2 marker CD206. This suggests a potential modulation of macrophage polarization balance towards an anti-inflammatory phenotype. Both transcriptomic and protein analyses confirmed that Paeoniflorin suppressed activation of the TLR4/NF-κB signaling pathway. The protective effects of Paeoniflorin were completely abolished by co-administration of the TLR4 agonist RS09 TFA.

**Conclusion:**

Paeoniflorin confers protection against sepsis-induced liver injury by modulating macrophage polarization from the pro-inflammatory M1 phenotype toward the anti-inflammatory M2 phenotype through inhibition of the TLR4/NF-κB signaling pathway. These findings identify Paeoniflorin as a promising candidate for further development as an immunomodulatory therapy for SALI.

## 1 Introduction

Sepsis, a life-threatening organ dysfunction caused by a dysregulated host response to infection, represents a major global health burden with high mortality rates in intensive care units (1, 2). The liver, as a central immune organ, plays a critical role in the host defense against pathogens and is highly vulnerable to sepsis-associated damage (3). Sepsis-associated liver injury (SALI) is a frequent and severe complication, occurring in approximately 20–50% of sepsis cases, and its development is independently associated with markedly increased mortality (4). Current therapeutic strategies for sepsis primarily focus on early antimicrobial therapy, source control, and supportive organ care (5, 6). However, these approaches lack targeted interventions to protect the liver from the uncontrolled inflammatory cascade that characterizes the septic response, underscoring an urgent need for novel hepatoprotective agents.

The pathophysiology of SALI involves a complex interplay of inflammatory mediators and immune cell dysregulation (7). Within the hepatic microenvironment, macrophages exert a dual influence, serving as both perpetrators and regulators of injury (8). In response to pathogen-associated molecular patterns (PAMPs) and damage-associated molecular patterns (DAMPs), macrophages can polarize into a pro-inflammatory M1 phenotype, characterized by the release of cytokines such as tumor necrosis factor-alpha (TNF-α), interleukin-6 (IL-6), and interleukin-1 beta (IL-1β) (9). This M1-driven “cytokine storm” contributes to hepatocyte apoptosis, microcirculatory dysfunction, and eventual organ failure (10). Conversely, the alternative M2 polarization state promotes inflammation resolution, tissue repair, and restoration of homeostasis (11). The dynamic balance between M1 and M2 macrophages is therefore a critical determinant of hepatic outcomes in sepsis, making the modulation of macrophage polarization a promising therapeutic strategy.

The Toll-like receptor 4 (TLR4)/nuclear factor-kappa B (NF-κB) signaling axis is a principal pathway driving M1 polarization and the subsequent inflammatory injury (12). TLR4 activation by ligands such as lipopolysaccharide (LPS) triggers a downstream cascade that leads to NF-κB translocation into the nucleus, initiating the transcription of pro-inflammatory genes (13, 14). Pharmacological inhibition of this pathway has been shown to attenuate excessive inflammation and ameliorate organ injury in experimental sepsis, highlighting its therapeutic relevance (15, 16). However, the clinical translation of synthetic TLR4/NF-κB inhibitors has been hampered by challenges related to specificity and side effects, shifting research interest towards naturally derived compounds with favorable safety profiles and multi-target potential.

Paeoniflorin, a primary bioactive monoterpene glycoside isolated from the roots of *Paeonia lactiflora* Pall., has garnered attention for its anti-inflammatory, immunoregulatory, and organ-protective properties in various disease models (17–19). Previous studies have reported its beneficial effects in conditions such as cerebral ischemia, rheumatoid arthritis, and hepatic fibrosis, often attributing these effects to the suppression of inflammatory signaling (20–23). Notably, emerging evidence suggests that Paeoniflorin can influence macrophage function and polarization. Nevertheless, its specific role and mechanism of action in the context of SALI, particularly concerning the regulation of macrophage polarization via the TLR4/NF-κB pathway, remain incompletely elucidated.

Based on this background, the current study aimed to evaluate the therapeutic potential of Paeoniflorin against SALI and elucidate its underlying mechanisms. Using a cecal ligation and puncture (CLP) murine model combined with transcriptomic and molecular analyses, we tested the hypothesis that Paeoniflorin protects against liver injury by promoting macrophage polarization from the M1 to the M2 phenotype via suppression of the TLR4/NF-κB pathway. Our findings provide mechanistic evidence supporting Paeoniflorin’s further development as an immunomodulatory agent for sepsis-induced liver injury.

## 2 Materials and methods

### 2.1 Mouse model establishment

105 male C57BL/6 mice (8 weeks old, 20–25 g) were obtained from the Tongji University Animal Center (Shanghai) and housed in individually ventilated cages under controlled conditions (20–22°C, 12-h light/dark cycle) with free access to standard chow and water. All experimental procedures involving animals were approved by the Institutional Animal Care and Use Committee of Tongji University (Approval No. TJBB07324108). The study was conducted in accordance with the National Institutes of Health Guide for the Care and Use of Laboratory Animals and the ARRIVE 2.0 guidelines. All efforts were made to minimize animal suffering and to use the minimum number of animals necessary for obtaining reliable data. Following a one-week acclimatization period, mice were randomly assigned to one of the following groups (n = 15 per group): Sham, CLP, CLP + Paeoniflorin (30, 60, or 120 mg/kg; SM5144, Beyotime), CLP + Paeoniflorin (120 mg/kg)+ TLR4 agonist (RS09 TFA, HY-D1056; 10 μg/kg), and Paeoniflorin-only (120 mg/kg). Paeoniflorin was administered daily via oral gavage for seven days, while RS09 TFA was injected intraperitoneally once daily over the same period. Paeoniflorin was dissolved in physiological saline (or 0.5% carboxymethylcellulose sodium) and administered daily via oral gavage at a volume of 10 mL/kg body weight for seven consecutive days.

Sample size determination and power analysis:

The sample size for animal experiments was determined based on prior experience with the CLP model in our laboratory and comparable studies in the literature investigating hepatoprotective agents in sepsis (24). For key continuous outcome measures (e.g., serum ALT/AST), a pilot study (n=3 per group) indicated an expected effect size (Cohen’s *d*) of approximately 2.0 between CLP and treatment groups. Using G*Power software (version 3.1), with an alpha level of 0.05 and a desired power (1 - β) of 0.80, the estimated sample size required per group for a two-tailed t-test was 5–6 animals. To account for potential mortality in the CLP group, ensure robust statistical analysis in multi-group comparisons (ANOVA), and provide sufficient tissue for multiple downstream assays, we increased the sample size to n = 15 per group for survival studies and n = 6 per group for biochemical and molecular analyses (randomly selected from survivors). This sample size is consistent with widely accepted standards in experimental sepsis research.

**Figure 1:**
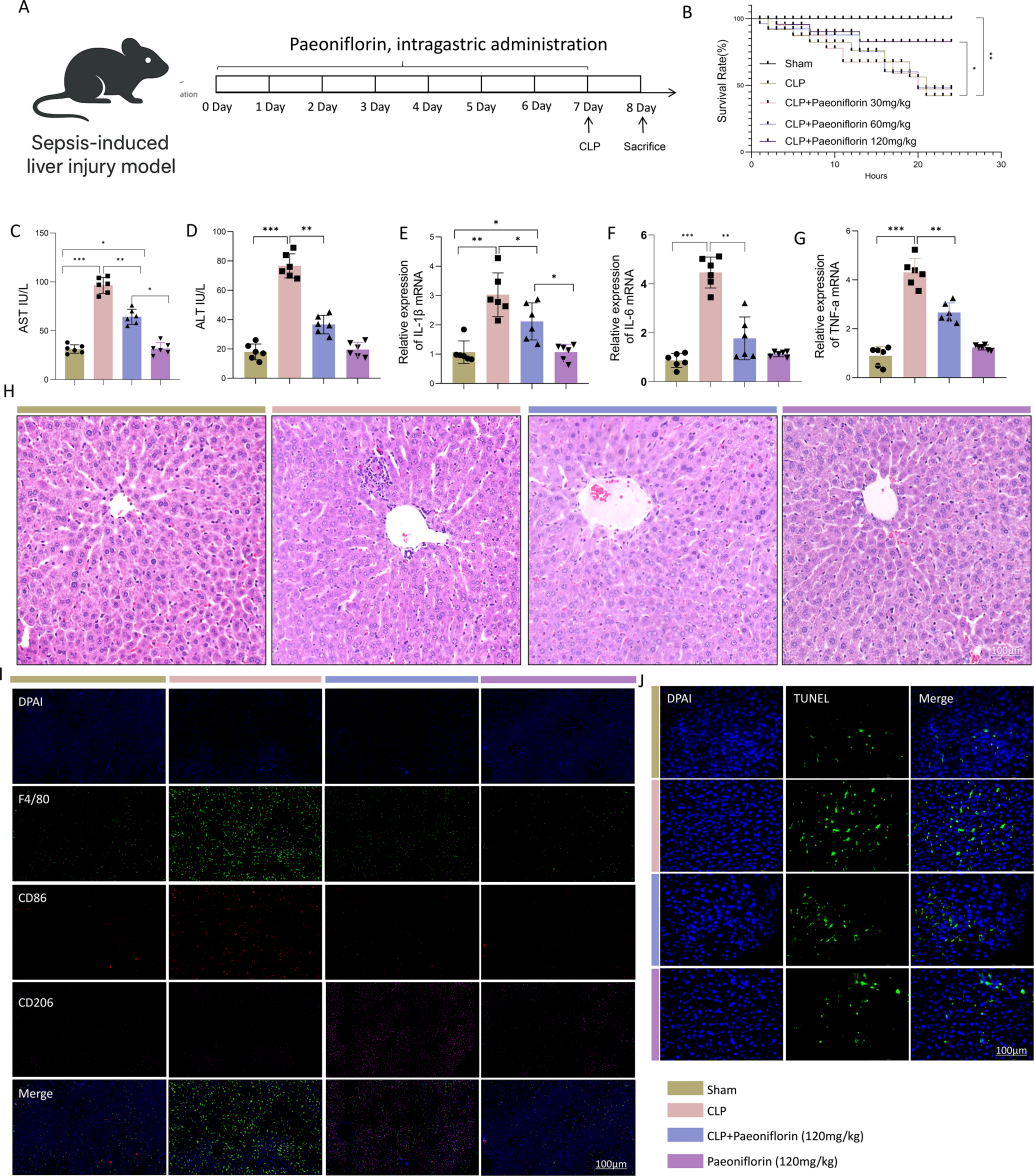
PaeoniflorinPaeoniflorin attenuates sepsis-induced liver injury by promoting macrophage polarization toward the M2 phenotype. **(A)** Schematic of the experimental timeline for sepsis induction by CLP and Paeoniflorin administration. **(B)** Kaplan-Meier survival curves of mice in different groups over 24 hours (n=15 per group).Statistical significance was determined by the log-rank test. **(C, D)** Serum levels of ALT and AST in Sham, CLP, CLP+Paeoniflorin and Paeoniflorin groups. **(E–G)** Hepatic mRNA expression levels of pro-inflammatory cytokines Il-1β, Il6, and Tnf-α measured by qRT-PCR. **(H)** Representative images of H&E-stained liver sections. Scale bar, 100 μm (n=3/group). **(I)** Representative immunofluorescence images of liver sections stained for F4/80(macrophages, green), CD86 (M1 marker, red), and CD206 (M2 marker, pink). Nuclei were counterstained with DAPI (blue) (n=3/ group). Scalebar, 100mm. **(J)** Representative images of TUNEL staining (green) for apoptotic hepatocytes. Nuclei were counterstained with DAPI (blue) (n=3/group). Scale bar, 100mm. Data in panels **(C–G)** are presented as mean ± SEM (n = 6/group, randomly selected from survivors for statistical comparability). Statistical significance was determined by one-way ANOVA with appropriate *post-hoc* tests.*P < 0.05, **P < 0.01, ***P<0.001.

On day 7, polymicrobial sepsis was induced by CLP. Briefly, mice were anesthetized with sodium pentobarbital (50 mg/kg, i.p, Sigma-Aldrich, P3761), and a midline laparotomy was performed under aseptic conditions. The cecum was exposed, ligated just distal to the ileocecal valve without causing intestinal obstruction, and then punctured through-and-through with a 21-gauge needle. A small amount of fecal content was extruded to ensure patency. The cecum was returned to the abdominal cavity, and the incision was closed in layers. Sham-operated mice underwent the same surgical procedure except for cecal ligation and puncture. Twenty-four hours after surgery, mice were euthanized by intraperitoneal injection of an overdose of sodium pentobarbital (150 mg/kg). Tissue and serum samples were collected for further analysis (**Figure 1A**).

### 2.2 Hepatic histopathology

Liver tissues were immersion-fixed in 10% neutral-buffered formalin for 24 h, followed by paraffin embedding. Sections of 4 μm thickness were prepared and stained with hematoxylin and eosin (H&E) for morphological evaluation. Pathological assessment was performed under a light microscope (Olympus CX30, Japan), with particular emphasis on alterations in lobular architecture, the extent of inflammatory cell infiltration, and other structural abnormalities.

### 2.3 qPCR

Liver specimens were snap-frozen in liquid nitrogen and stored at –80°C until use. Total RNA was isolated using Trizol reagent (15596026, Invitrogen, USA) according to the manufacturer’s instructions. cDNA was synthesized from equal amounts of RNA and subsequently amplified using the Universal SYBR FAST qPCR Kit Master Mix (KAPA Biosystems, USA) on a real-time PCR system. The thermocycling protocol consisted of an initial denaturation at 95°C for 10 min, followed by 45 cycles of 95°C for 10 s, 59°C for 60 s, and 72°C for 15 s, with a final extension at 95°C for 15 s. The following primer sequences (Invitrogen, Shanghai) were used for amplification:

TNF-α:
F: 5′-GTTCTATGGCCCAGACCCTCAC-3′
R: 5′-GGCACCACTAGTTGGTTGTCTTTG-3′
IL-1β:
F: 5′-TCCAGGATGAGGACATGAGCAC-3′
R: 5′-GAACGTCACCCAGCAGGTTA-3′
IL-6:
F: 5′-CCACTTCACAAGTCGGAGGCTTA-3′
R: 5′-CCAGTTTGGTAGCATCCATCATTTC-3′
GAPDH (internal control):
Forward: 5′-AGGTCGGTGTGAACGGATTTG-3′
Reverse: 5′-TGTAGACCATGTAGTTGAGGTCA-3′

Gene expression levels were normalized to GAPDH and analyzed using the 2^^–ΔΔCt^ method.

### 2.4 Western blotting

Proteins were extracted from homogenized liver tissue using RIPA lysis buffer containing protease and phosphatase inhibitors. Protein concentrations were determined with a BCA assay kit (Beyotime, China). Equal amounts of protein (30 μg per lane) were separated by 10% SDS-PAGE using the Bio-Rad Mini-PROTEAN system and subsequently transferred onto PVDF membranes. After blocking with 5% non-fat milk in TBST for 1 h at 37°C, the membranes were incubated overnight at 4°C with the following primary antibodies: anti-TLR4 (Abcam, ab13556, 1:1000), anti-NF-κB (Immunoway, #YT5382, 1:1000), anti-phospho-NF-κB (CST, #67824, 1:1000), and anti-GAPDH (Proteintech, #60004-1-Ig, 1:5000). Following three washes with TBST, the membranes were incubated with corresponding HRP-conjugated secondary antibodies (Beyotime or Proteintech, 1:2000) for 1 h at room temperature. Protein bands were visualized using an enhanced chemiluminescence (ECL) detection system, and band intensities were quantified by densitometry using ImageJ software (v1.50i). GAPDH served as the internal loading control for normalization.

### 2.5 Immunohistochemistry

Paraffin-embedded sections were deparaffinized in xylene and rehydrated through a graded ethanol series. Antigen retrieval was performed by heat-induced epitope retrieval in citrate buffer (pH 6.0) using a pressure cooker. Endogenous peroxidase activity was quenched by incubation with 3% hydrogen peroxide for 15 minutes, and nonspecific binding sites were blocked with 5% bovine serum albumin for 20 minutes at room temperature. The sections were subsequently incubated overnight at 4°C with the following primary antibodies: anti-CD206 (CST, #E6T5J, 1:500), anti-CD86 (CST, #E5W6H, 1:500), and anti-F4/80 (Wanlei, #WLH2545, 1:500). After washing, the sections were incubated with HRP-conjugated secondary antibody (Beyotime, 1:700) for 1 hour at room temperature. Immunoreactivity was visualized using 3, 3’-diaminobenzidine (DAB) substrate, followed by counterstaining with hematoxylin. Finally, all slides were digitally scanned using a high-resolution slide scanner for subsequent analysis.For quantitative analysis, five non-overlapping high-power fields (HPF, 400× magnification) were randomly selected per section. All quantifications were performed by two independent investigators who were blinded to the experimental group allocation. Positive cells were counted manually, or the integrated optical density was measured using ImageJ software (v1.50i) with consistent threshold settings applied across all samples. The average value from the two investigators was used for statistical analysis.

### 2.6 Immunohistochemical staining

Following deparaffinization and rehydration, antigen retrieval was carried out using citrate buffer (pH 6.0) via heat-induced epitope retrieval. Tissue sections were then treated with 3% H_2_O_2_ to block endogenous peroxidase activity, followed by blocking with 5% BSA as described previously. The sections were incubated overnight at 4°C with primary antibodies targeting either NF-κB (Immunoway, #YT5382, 1:1000) or phosphorylated NF-κB (CST, #67824, 1:1000). After thorough washing, HRP-conjugated secondary antibodies were applied for 1 h at room temperature. Signal detection was performed using a DAB substrate kit (Maxim), followed by counterstaining with hematoxylin. Finally, the stained sections were dehydrated, mounted with neutral resin, and digitally scanned for subsequent analysis.

### 2.7 TUNEL staining

Apoptotic cells in liver tissues were detected using a TUNEL apoptosis detection kit (Beyotime, #C1088) according to the manufacturer’s protocol. Briefly, after deparaffinization and rehydration, tissue sections were fixed with 1% paraformaldehyde for 15 min at room temperature and then treated with proteinase K (20 μg/mL) for 10 min at 37°C. Sections were subsequently incubated with the TUNEL reaction mixture for 60 min at 37°C in a dark humidified chamber. Nuclei were counterstained with DAPI, and slides were mounted with anti-fade mounting medium (Solarbio). Five randomly selected fields per section were imaged using a fluorescence microscope (Olympus IX71) under 100× magnification. The number of TUNEL-positive cells was quantified using ImageJ software and expressed as a percentage of the total DAPI-stained nuclei.

### 2.8 Liver testing

Serum levels of alanine aminotransferase (ALT) and aspartate aminotransferase (AST) were quantified using commercial assay kits (ALT, C009-2-1; AST, C010-2-1; Nanjing Jiancheng Bioengineering Institute, China) according to the manufacturer’s instructions. Measurements were performed on an Olympus AU400 automated biochemical analyzer. Enzyme activities were expressed as units per liter (U/L).

### 2.9 RNA sequencing

Total RNA was extracted from liver tissues collected from septic and Paeoniflorin-treated septic mice (n = 4 per group). RNA integrity was verified using an Agilent 2100 Bioanalyzer, and sequencing libraries were constructed following the manufacturer’s instructions. The libraries were sequenced on an Illumina NovaSeq 6000 platform (Novogene, Beijing) to generate 150 bp paired-end reads. After quality control and adapter trimming, clean reads were aligned to the murine reference genome (GRCm39) using HISAT2 (v2.0.5). Gene-level counts were quantified with featureCounts (v1.5.0-p3), and expression levels were normalized as fragments per kilobase of transcript per million mapped reads (FPKM). Differential expression analysis was performed using DESeq2, with significance defined as an absolute log_2_ fold change > 1.5 and a Benjamini-Hochberg adjusted *p*-value (FDR) < 0.05 to control for multiple testing. This approach rigorously accounts for the multiple testing burden across thousands of genes. Genes meeting these criteria were defined as differentially expressed genes (DEGs) and subjected to KEGG pathway enrichment analysis to identify significantly altered biological pathways.

### 2.10 Statistical analyses

All data are expressed as the mean ± standard error of the mean (SEM). The normality of data distribution was assessed using the Shapiro–Wilk test. For comparisons among multiple groups: One-way analysis of variance (ANOVA) was employed, followed by Tukey’s honestly significant difference (HSD) *post-hoc* test for multiple comparisons. Tukey’s test controls the family-wise error rate across all pairwise comparisons within the ANOVA model. Differences between two groups under normal distribution were analyzed using Student’s t-test. For data that did not meet the normality assumption, the Kruskal–Wallis test (multiple groups) or Mann–Whitney U test (two groups) was applied accordingly. A two-tailed P value of less than 0.05 was considered statistically significant.

## 3 Results

### 3.1 Paeoniflorin attenuates sepsis-induced liver injury by promoting macrophage M2 polarization

To investigate the hepatoprotective mechanisms of Paeoniflorin in SALI, we first assessed key liver injury markers and immune responses. Survival analysis performed using the Kaplan-Meier method revealed that the CLP group exhibited a significantly lower survival probability within 24 hours compared to the Sham group. Paeoniflorin(120mg/kg) administration, however, markedly improved the survival rate of septic mice (*p* < 0.05 vs. CLP group, log-rank test). (**Figure 1B**). Mice subjected to CLP showed significantly elevated serum levels of ALT and AST, which were dose-dependently reduced by Paeoniflorin treatment (**Figures 1C, D**). Consistent with these findings, qPCR analysis revealed that CLP surgery markedly upregulated the mRNA expression of pro-inflammatory cytokines (IL-1β, IL-6, and TNF-α) compared to the Sham group, whereas Paeoniflorin administration substantially suppressed these increases (**Figures 1E–G**). Histopathological evaluation of H&E-stained liver sections from CLP mice displayed severe lobular disorganization, edema, inflammatory cell infiltration, and hemorrhage. These structural abnormalities were notably mitigated in the Paeoniflorin-treated groups (**Figure 1H**). Immunofluorescence analysis further demonstrated extensive infiltration of F4/80^+^CD86^+^ M1 macrophages in CLP-induced septic livers. In contrast, Paeoniflorin promoted a significant increase in F4/80^+^CD206^+^ M2 macrophages, indicating a shift in macrophage polarization from a pro-inflammatory M1 to an anti-inflammatory M2 phenotype (**Figure 1I**). In addition, TUNEL staining revealed substantial hepatocyte apoptosis in septic mice, which was markedly attenuated following Paeoniflorin intervention (**Figure 1J**). Taken together, these results demonstrate that Paeoniflorin effectively ameliorates sepsis-induced liver injury, at least in part through reprogramming of the hepatic immune microenvironment via promotion of M2 macrophage polarization.

### 3.2 Paeoniflorin ameliorates sepsis-induced liver injury via modulation of the NF-κB signaling pathway

**Figure 2:**
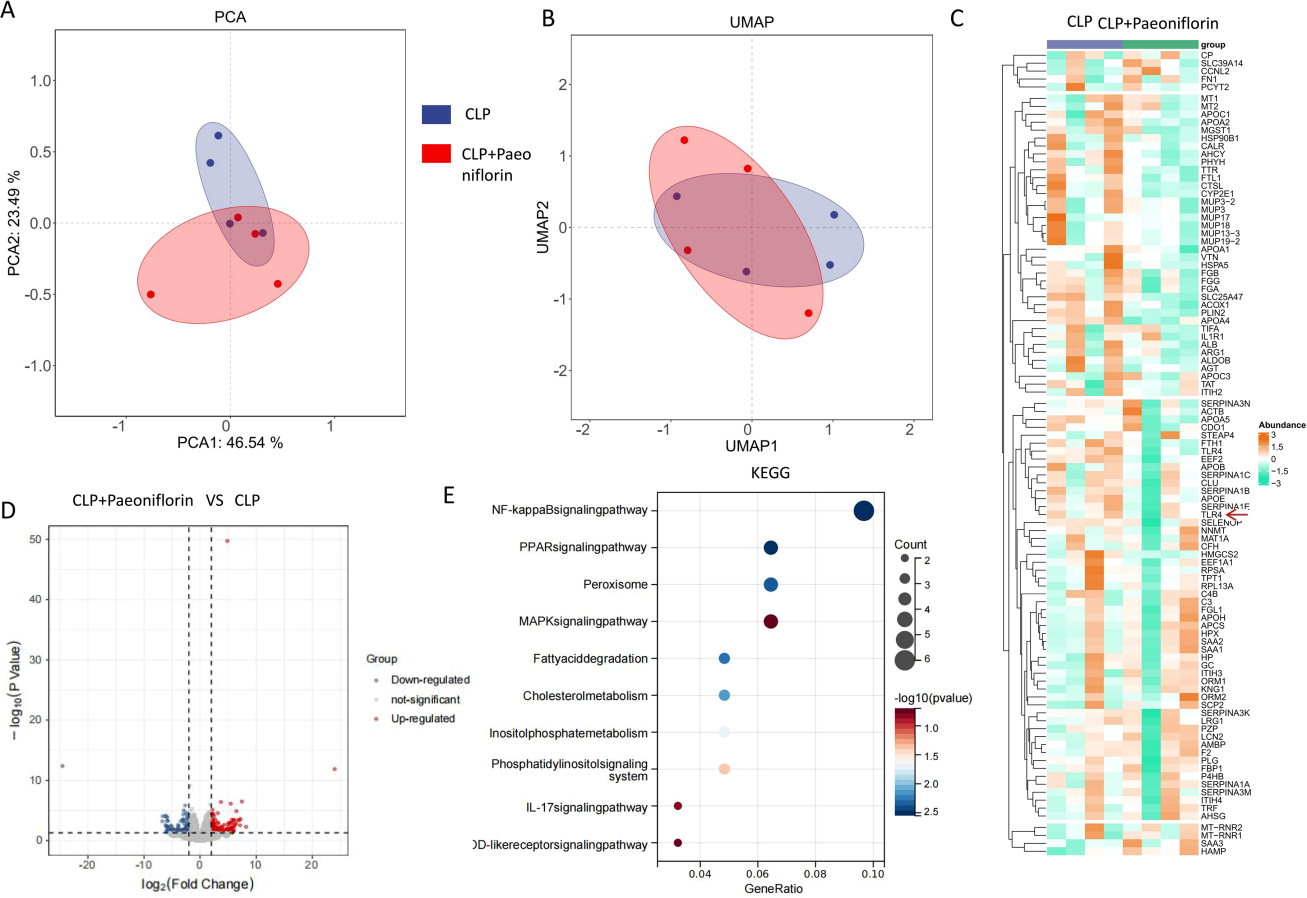
Transcriptomic analysis reveals PaeoniflorinPaeoniflorin-mediated suppression of the NF-κB signaling pathway. **(A)** Principal component analysis (PCA) plot of RNA-seq data from CLP and CLP+PaeoniflorinPaeoniflorin groups. **(B)** UMAP visualization showing distinct clustering of transcriptomic profiles between experimental groups. **(C)** Heatmap of the top 50 DEGs between treatment groups.Paeoniflorin **(D)**Volcano plot displaying differentially expressed genes (DEGs) between CLP and CLP+Paeoniflorin groups. Red points indicate significantly upregulated genes, blue points indicate downregulated genes (|log_2_FC| > 1.5, adjusted *p* < 0.05). **(E)** KEGG pathway enrichment analysis of DEGs, showing the NF-κB signaling pathway as the most significantly enriched. Data are presented as mean ± SEM (n = 4/group). *P<0.05, **P<0.01, ***P<0.001.

To further elucidate the molecular mechanisms underlying the protective effect of Paeoniflorin, we performed transcriptomic profiling of liver tissues from CLP-induced septic mice with or without Paeoniflorin treatment (n=4 per group). Principal component analysis (PCA) and uniform manifold approximation and projection (UMAP) of the global transcriptome demonstrated clear separation between the CLP and CLP+Paeoniflorin groups, indicating distinct transcriptomic profiles (**Figures 2A, B**). Heatmap of the top 50 DEGs between treatment groups (**Figure 2C**). Differential gene expression analysis identified numerous significantly altered genes, visualized by volcano plots, which revealed extensive transcriptional reprogramming in response to sepsis (**Figure 2D**). Kyoto Encyclopedia of Genes and Genomes (KEGG) pathway enrichment analysis further identified the NF-κB signaling pathway as the most significantly upregulated pathway in the CLP group (**Figure 2E**), and its was also found that TLR4 was higher expressed in the CLP group compared with the Paeoniflorin treatment group (**Figure 2C**). Collectively, these transcriptomic findings indicate that Paeoniflorin alleviates sepsis-associated liver injury by suppressing TLR4/NF-κB signaling activation, thereby restraining pro-inflammatory M1 macrophage polarization.

### 3.3 TLR4 inhibition is essential for the hepatoprotective effects of paeoniflorin

**Figure 3:**
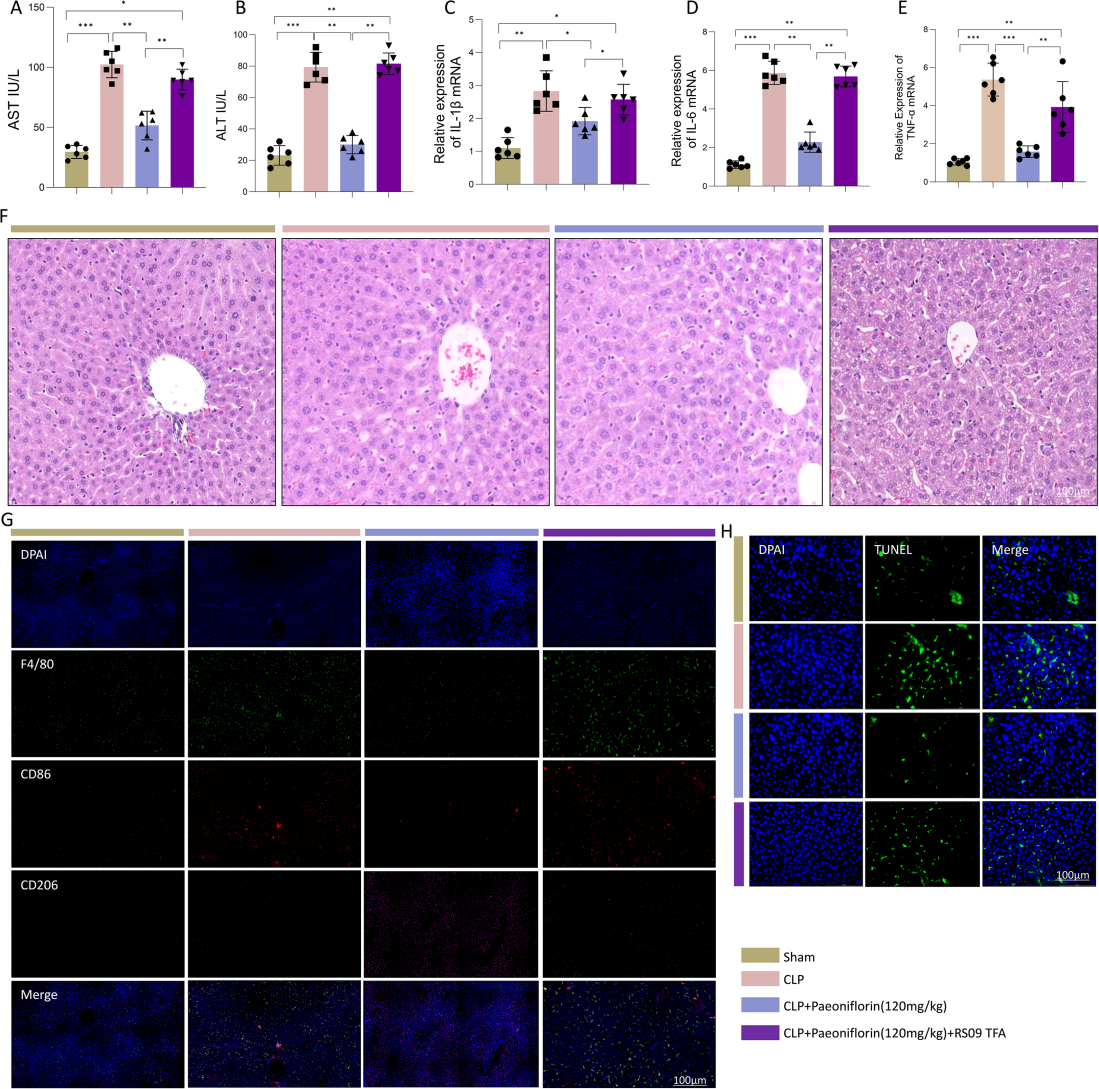
TLR4 activation reverses the protective effects of PaeoniflorinPaeoniflorin against sepsis-induced liver injury. **(A, B)** Serum **(A)** AST and **(B)** ALT levels in Sham, CLP, CLP+PaeoniflorinPaeoniflorin, and CLP+PaeoniflorinPaeoniflorin+RS09 TFA groups. **(C–E)** Hepatic mRNA expression of **(C)** IL-1β, **(D)** IL-6, and **(E)**TNF-α.(n=6/group) **(F)** Representative H&E-stained liver sections (n=3/group) (scale bar = 100 μm). **(G)** Immunofluorescence staining for F4/80, CD86, and CD206 in liver tissues (n=3/group) (scale bar = 100 μm). **(H)** TUNEL staining showing apoptotic cells (green) with DAPI counterstain (blue) (n=3/group) (scale bar = 100 μm). Statistical significance was determined by one-way ANOVA. Data are presented as mean ± SEM. *P<0.05, **P<0.01, ***P<0.001.

To determine whether TLR4 signaling mediates the hepatoprotective actions of Paeoniflorin, we evaluated key liver injury parameters under conditions of TLR4 pharmacological activation. Consistent with our earlier observations, Paeoniflorin treatment significantly attenuated CLP-induced elevations in serum AST and ALT levels; however, co-administration of the TLR4 agonist RS09 TFA completely reversed this protective effect, resulting in renewed transaminase elevation (**Figures 3A, B**). Correspondingly, qPCR analysis demonstrated that Paeoniflorin substantially suppressed the CLP-induced mRNA expression of pro-inflammatory cytokines (IL-6, TNF-α, and IL-1β), while RS09 TFA co-treatment restored their overexpression (**Figures 3C–E**). Histopathological examination via H&E staining revealed that RS09 TFA administration exacerbated lobular architecture disruption, edema, and inflammatory cell infiltration, in contrast to the well-preserved hepatic structure in the Paeoniflorin-treated group (**Figure 3F**). Immunofluorescence analysis further indicated that Paeoniflorin promoted a shift in macrophage polarization, characterized by reduced F4/80^+^CD86^+^ M1 macrophage accumulation and enhanced F4/80^+^CD206^+^ M2 macrophage infiltration. This effect was abrogated by RS09 TFA co-treatment (**Figure 3G**). Additionally, TUNEL assay confirmed that Paeoniflorin significantly reduced CLP-induced hepatocyte apoptosis, an anti-apoptotic effect that was nullified upon TLR4 activation (**Figure 3H**). Taken together, these findings demonstrate that TLR4 inhibition is indispensable for the protective role of Paeoniflorin against sepsis-associated liver injury.

### 3.4 Paeoniflorin confers its protective effects through suppression of the TLR4/NF-κB signaling axis

**Figure 4:**
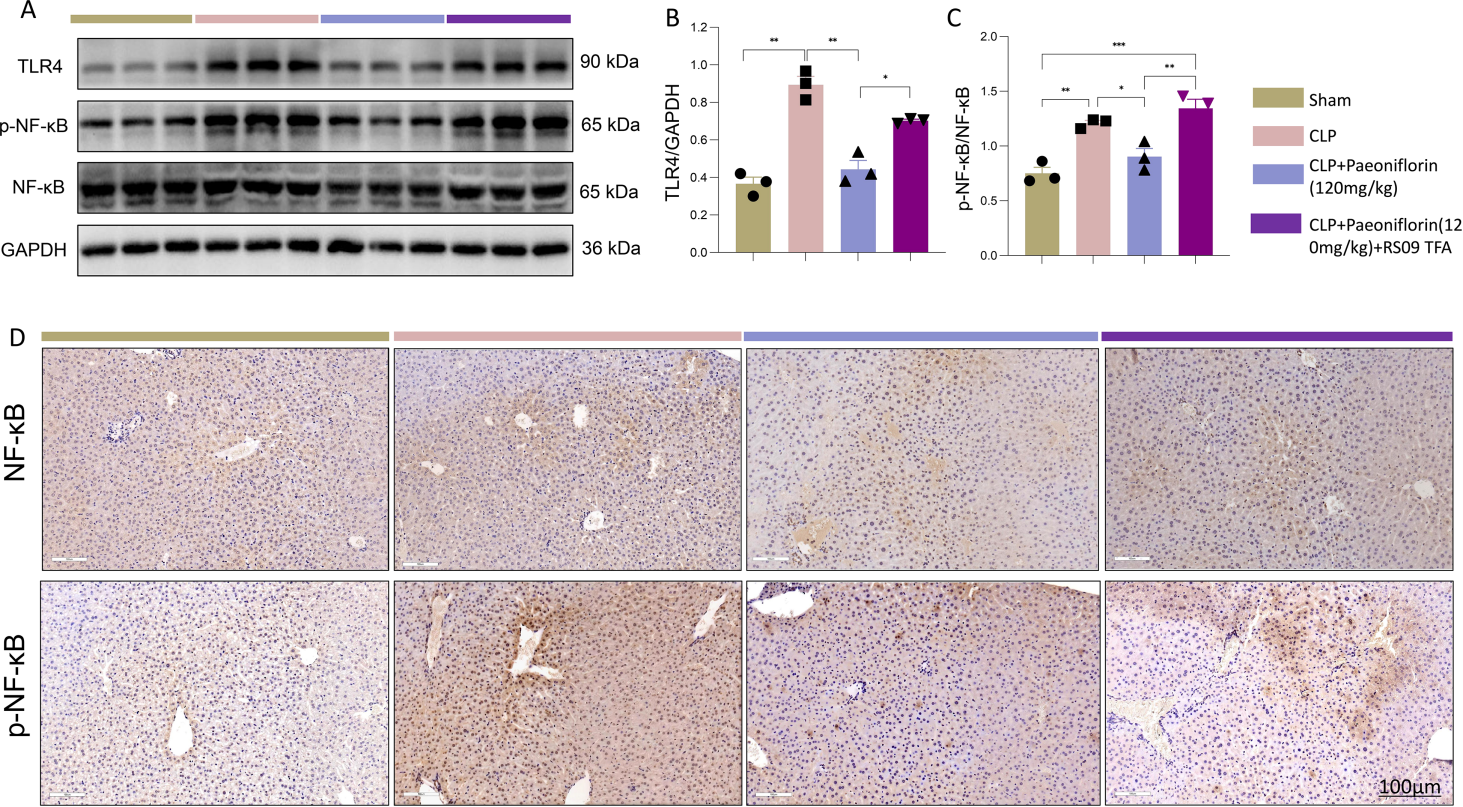
PaeoniflorinPaeoniflorin inhibits the TLR4/NF-κB signaling pathway in septic liver injury. **(A)** Representative Western blot bands of TLR4, NF-κB p65, phosphorylated NF-κB p65 (p-p65), and GAPDH. **(B, C)** Densitometric quantification of **(B)** TLR4, **(C)** p NF-κB/total NF-κB protein levels normalized to GAPDH. **(D)** Immunohistochemical staining of NF-κB and p NF-κB in liver sections (scale bar = 100 μm). Statistical significance was determined by one-way ANOVA. Data are presented as mean ± SEM (n = 3/group). *P<0.05, **P<0.01, ***P<0.001.

Given that TLR4 activation ultimately leads to NF-κB pathway induction, we next investigated whether Paeoniflorin modulates this downstream signaling cascade. Sepsis induction resulted in significantly increased hepatic protein levels of both total NF-κB p65 and its phosphorylated form (p-p65). Paeoniflorin treatment effectively suppressed the expression of total and phosphorylated NF-κB, whereas concurrent administration of the TLR4 agonist RS09 TFA restored their elevated expression (**Figures 4A–E**). Immunohistochemical analysis corroborated these findings, demonstrating strong nuclear staining of p65 and p-p65 in septic liver tissues, which was markedly reduced by Paeoniflorin treatment but re-established upon RS09 TFA co-administration (**Figures 4F–G**). These consistent results across Western blot and immunohistochemical approaches confirm that Paeoniflorin alleviates sepsis-induced liver injury by specifically inhibiting TLR4-mediated NF-κB activation, thereby preventing pro-inflammatory M1 macrophage polarization and attenuating excessive inflammatory responses. Our findings establish the TLR4/NF-κB pathway as a crucial mechanistic target through which Paeoniflorin exerts its hepatoprotective effects in the context of sepsis.

## 4 Discussion

Sepsis-associated liver injury remains a significant contributor to mortality in critically ill patients, with current therapeutic strategies offering limited protection against progressive hepatic dysfunction (25–27). These findings established Paeoniflorin as a potent hepatoprotective agent in experimental sepsis, functioning through sophisticated immunomodulatory mechanisms that target both inflammatory signaling and cellular reprogramming. These results not only confirmed Paeoniflorin’s therapeutic potential but also provided new insights into macrophage biology in sepsis pathogenesis.

The robust hepatoprotection observed with Paeoniflorin treatment - manifested through improved biochemical parameters, preserved tissue architecture, and reduced apoptosis - represents a significant advancement in the search for effective SALI therapies. Particularly noteworthy is the compound’s ability to simultaneously mitigate multiple aspects of liver injury, suggesting a comprehensive protective mechanism. This multi-faceted protection aligns with recent paradigm shifts in sepsis management that emphasize the importance of addressing organ dysfunction through integrated approaches rather than isolated interventions (28).

Our findings position among other natural compounds known to modulate the TLR4/NF-κB pathway in sepsis, such as curcumin, resveratrol, and baicalin. While these compounds share the common theme of anti-inflammatory activity, Paeoniflorin may offer distinct advantages. As a monoterpene glycoside, its specific pharmacokinetic profile could differ. More importantly, beyond suppressing pro-inflammatory signaling, our data highlight Paeoniflorin’s potent capacity to actively drive the repolarization of hepatic macrophages from the M1 to the M2 phenotype. This dual action—simultaneously inhibiting a key inflammatory driver (TLR4/NF-κB) and promoting a pro-resolutive immune microenvironment—may provide a more comprehensive therapeutic strategy for SALI, targeting both injury propagation and tissue repair pathways.Paeoniflorin These findings gains additional significance in the context of recent single-cell RNA sequencing studies that have unveiled unprecedented heterogeneity in hepatic macrophage populations during sepsis (29). The specific enhancement of F4/80^+^/CD206^+^ M2 macrophages suggests that Paeoniflorin may be targeting particular macrophage subsets with specialized repair functions, rather than inducing global changes in macrophage populations.

The transcriptomic and molecular analyses provide compelling evidence that TLR4/NF-κB signaling served as the primary molecular target for Paeoniflorin. This study extended beyond simply identifying pathway inhibition to demonstrate the functional consequences of this suppression. The complete reversal of Paeoniflorin’s protective effects by RS09 TFA co-administration offers particularly strong validation of TLR4’s central role (30). These findings resonated with emerging research highlighting the complexity of TLR4 signaling in sepsis, including recent discoveries of tissue-specific regulation and novel downstream effectors (31). Interestingly, our observations align with cutting-edge research demonstrating that natural compounds can achieve selective TLR4 modulation through allosteric mechanisms that differ from synthetic inhibitors.

Recent advances in immunometabolism provided additional context for interpreting our results. The polarization of macrophages is now recognized to involve profound metabolic reprogramming, with M1 macrophages relying predominantly on glycolysis and M2 macrophages utilizing oxidative phosphorylation (32). While our study focused on signaling pathways, Paeoniflorin’s effects on macrophage polarization might involve modulation of these metabolic pathways, representing an exciting direction for future investigation. Additionally, the growing understanding of mitochondrial dynamics in immune cell function suggests another potential mechanism through which Paeoniflorin may exert its effects (33).

Several intriguing questions emerge from our findings. First, the precise molecular interactions between Paeoniflorin and the TLR4 receptor complex warrant detailed structural studies. Second, the potential cross-talk between Paeoniflorin’s immunomodulatory effects and its direct actions on hepatocytes merits investigation, especially given emerging evidence of bidirectional communication between parenchymal and immune cells in sepsis. Third, the impact of Paeoniflorin on newly identified regulators of macrophage function, including various non-coding RNAs and epitranscriptomic modifications, represents a promising research direction.

Our study also raised important considerations regarding the translational potential of Paeoniflorin. While the demonstrated efficacy in a well-established sepsis model is encouraging, the compound’s pharmacokinetic profile, optimal dosing regimen, and safety in the context of polymicrobial infection require comprehensive evaluation. Furthermore, the potential synergy between Paeoniflorin and conventional antibiotics represents an clinically relevant question that deserves attention, given the central role of antimicrobial therapy in sepsis management. The transcriptomic analysis in this study was designed to compare the CLP group with the CLP+Paeoniflorin group, which successfully identified key pathways influenced by the treatment. However, the absence of a Sham control group in the RNA-seq design limits the ability to distinguish sepsis-induced transcriptional changes from those specifically mediated by Paeoniflorin. Future studies incorporating a Sham group will help clarify these distinct effects. Additionally, it is important to note that Paeoniflorin was administered prophylactically for seven days before sepsis induction in this model. While this approach effectively highlighted its hepatoprotective and immunomodulatory properties, it does not replicate the clinical context of treating established sepsis. Further research evaluating Paeoniflorin administration after sepsis onset is needed to determine its therapeutic potential.

It is important to acknowledge a key methodological limitation in our characterization of macrophage polarization. Our conclusions are predominantly derived from analysis of CD86 and CD206—classic but broad markers for M1- and M2-like phenotypes, respectively. While these results consistently suggest a shift in inflammatory balance, they do not delineate the specific macrophage subpopulations involved (e.g., monocyte-derived macrophages versus tissue-resident Kupffer cells, or distinct subsets within the M2 spectrum). Thus, claims regarding the effect of Paeoniflorin on ‘specific macrophage subpopulations’ warrant further substantiation. Future investigations utilizing high-dimensional approaches, such as flow cytometry with expanded marker panels (e.g., CD80, CD163, CD301, MHC-II) or single-cell RNA sequencing, will be essential to comprehensively define the hepatic macrophage landscape and precisely identify the subsets modulated by Paeoniflorin during sepsis.

In conclusion, this work established Paeoniflorin as a sophisticated immunomodulator that protects against sepsis-induced liver injury through coordinated regulation of macrophage polarization and TLR4/NF-κB signaling. The findings contribute to the growing recognition that targeted immunomodulation, rather than broad immunosuppression, represents a promising approach to sepsis management.

## Data Availability

The datasets presented in this study can be found in online repositories. The names of the repository/repositories and accession number(s) can be found below: <b> https://ngdc.cncb.ac.cn/gsa, CRA033591</b>.
