# Supplementary figures and images for: Paeoniflorin attenuates sepsis-induced liver injury by reprogramming macrophage polarization via the TLR4/NF-κB pathway
Source: Front Immunol. 2026 Jan 20;16:1751550. doi: 10.3389/fimmu.2025.1751550 (PMC12864077; doi:10.3389/fimmu.2025.1751550)

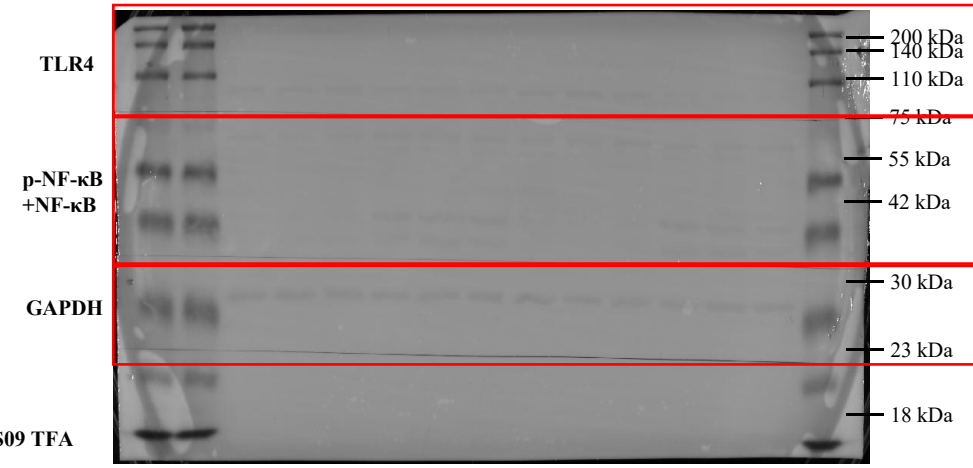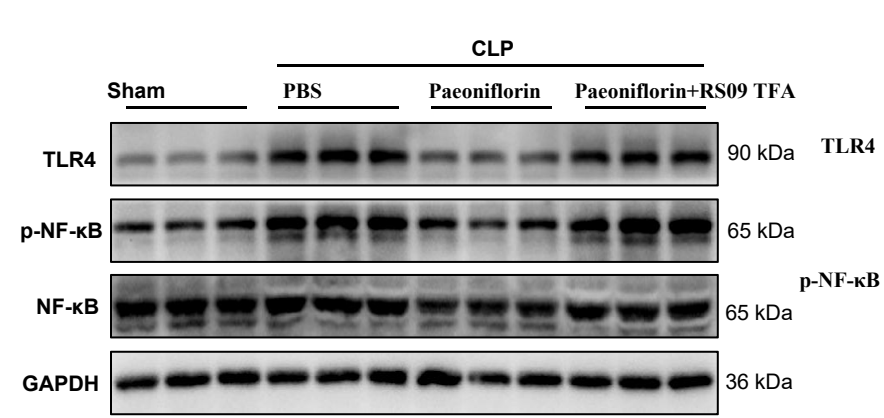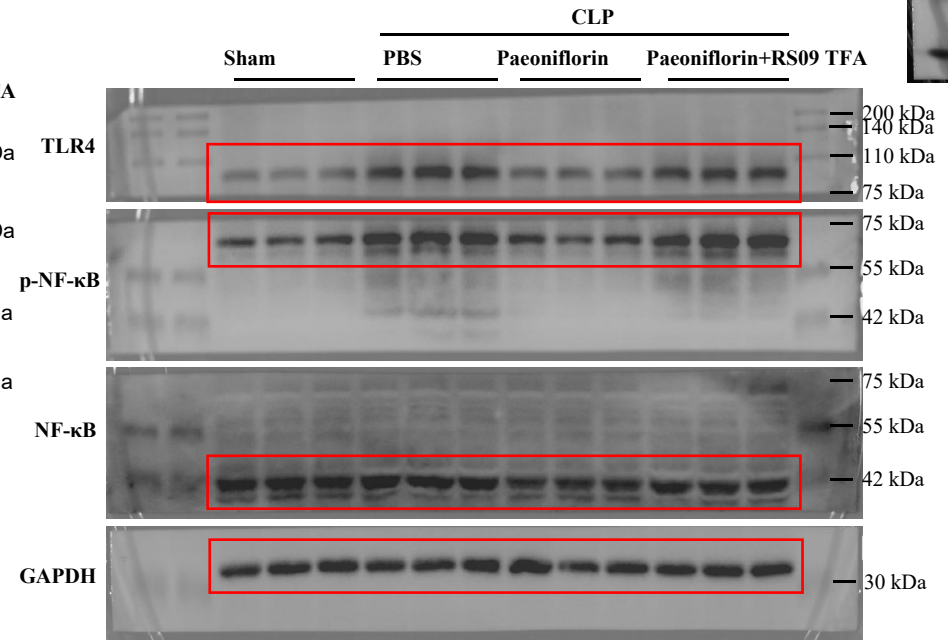

Supplement: Supplementary file 1 [file DataSheet1.pdf]
